# Fast reconstruction of EEG signal compression sensing based on deep learning

**DOI:** 10.1038/s41598-024-55334-9

**Published:** 2024-03-01

**Authors:** XiuLi Du, KuanYang Liang, YaNa Lv, ShaoMing Qiu

**Affiliations:** 1 School of Information Engineering, Dalian University, Dalian, 116622 China; 2 Communication and Network Laboratory, Dalian University, Dalian, 116622 China

**Keywords:** Compressed sensing, Residual networks, One-dimensional dilated convolution, EEG signals, Real-time reconfiguration, Computer science, Information technology

## Abstract

When traditional EEG signals are collected based on the Nyquist theorem, long-time recordings of EEG signals will produce a large amount of data. At the same time, limited bandwidth, end-to-end delay, and memory space will bring great pressure on the effective transmission of data. The birth of compressed sensing alleviates this transmission pressure. However, using an iterative compressed sensing reconstruction algorithm for EEG signal reconstruction faces complex calculation problems and slow data processing speed, limiting the application of compressed sensing in EEG signal rapid monitoring systems. As such, this paper presents a non-iterative and fast algorithm for reconstructing EEG signals using compressed sensing and deep learning techniques. This algorithm uses the improved residual network model, extracts the feature information of the EEG signal by one-dimensional dilated convolution, directly learns the nonlinear mapping relationship between the measured value and the original signal, and can quickly and accurately reconstruct the EEG signal. The method proposed in this paper has been verified by simulation on the open BCI contest dataset. Overall, it is proved that the proposed method has higher reconstruction accuracy and faster reconstruction speed than the traditional CS reconstruction algorithm and the existing deep learning reconstruction algorithm. In addition, it can realize the rapid reconstruction of EEG signals.

## Introduction

Compressed Sensing (CS), also known as Compressive Sampling, represents a significant breakthrough in the field of signal processing. Introduced by Donoho, Candes, Romberg, and Tao^1–3^, CS is applicable in several domains where: (i) the number of sensors is limited due to high costs, such as non-visible wavelengths; (ii) measurement costs are prohibitive, exemplified by high-speed A/D converters used in neutron scattering imaging; (iii) sensing is time-consuming, like in medical imaging; and (iv) there are power limitations for sensing, etc.^4–7^. The current trend in CS involves its integration with bioelectric potential signals for compressive sensing or sampling. Performance metrics such as the Root Mean Square Deviation (RMSD) are utilized to objectively quantify the functionality of CS. Furthermore, CS is associated with state-of-the-art compression algorithms for Electrocardiograms (ECG) and Electroencephalograms (EEG) used in modern healthcare^8^.

As medical care continues to improve, early diagnosis of epilepsy or stroke patients through analyzing the EEG signal can help prevent exacerbations and provide timely treatment^9^. In addition, patients require remote monitoring of their health status during hospitalization, and physicians can conveniently address the recurrence of their condition by quickly reconstructing the EEG signal in the office, thus effectively improving the efficiency of medical resources utilization. Currently, with the increasing population aging and the growing number of patients with brain disorders, there is an ever-increasing emphasis on rapid health status monitoring. In particular, there is a need to provide remote monitoring facilities to populations living in rural areas, where medical facilities have limited coverage and patients may not receive timely treatment during morbidity. For remote monitoring, subjects need to wear some sensor nodes to acquire EEG signals, which are transmitted instantaneously to a remote facility. In this process, the patient is monitored by analyzing the acquired signals. Furthermore, data storage and electronic medical records^10^ are also important components of remote monitoring. These contents can be stored through cloud storage and other methods for doctors and patients to access and share at any time. However, the wireless transmission component consumes the most energy in an environment where EEG signals are continuously monitored over a long period of time^11^, where the low-energy transmission cannot be achieved using the traditional Nyquist sampling method. Compressive Sensing^11^ theory has been proposed to solve this problem by sampling the signal at a frequency much lower than the Nyquist sampling frequency and projecting the signal to a lower dimension through a simple matrix–vector product. Accordingly, it allows for achieving the goal of reducing transmission power consumption by compressing the signal before transmission and being able to retain important diagnostic information. Moreover, on the storage end, compressive sensing technology can compress large amounts of EEG data into smaller sizes, thus saving storage space.

Brain-Computer Interfaces (BCI)^12^: BCI is a technology that enables interaction with computers or external devices by directly interpreting brain activity. CS can be employed to reduce the dimensionality and computational load required to extract features from EEG signals, thereby enhancing the real-time performance and efficiency of BCI systems. EEG data typically contains a large volume of time-series data, which occupies significant storage space and transmission bandwidth. CS can be utilized to decrease the storage and transmission overhead of EEG data. By compressing EEG signals, they can be transformed into a more compact representation, thus reducing the demand for storage space and transmission bandwidth.

The process of EEG signal compression perception is generally divided into two steps. To begin with, at the acquisition end, a fixed sensing matrix is utilized to capture and compress the EEG signal, which is then transmitted to a remote facility in its compressed form. Since CS is a simple linear operation, the computational complexity at the acquisition end is low^13^. Second, reconfiguration algorithms reconstruct the received compressed signals at the remote facility. Reconstruction algorithms are divided into three main categories: greedy algorithms^14^, convex optimization algorithms^15^, and Bayesian learning algorithms^16^. Typically, the above algorithms iteratively reconstruct the original signal by solving the optimization problem based on sparse prior knowledge of the signal^17^. However, iterative reconstruction algorithms are computationally complex and time-consuming. In many cases, delays in reconstruction are intolerable. For example, Mohammad H. Aghababaei^18^ proposed a new feature for real-time automatic single-channel epilepsy detection based on the iterative application of the Orthogonal Matching Pursuit (OMP) algorithm to compressed EEG data to calculate the energy increase rate of the partially reconstructed signal. The feature Partial Energy Difference (PED) is then used to classify epileptic and non-epileptic seizure states. Results show that the proposed features can differentiate between epileptic and non-epileptic seizure periods even with a compression ratio (CR) as small as 0.05^18^. But the time-consuming nature does not allow clinicians in telemedicine facilities to diagnose the patient’s condition in a timely manner.

Based on the above problem, Angshul Majumdar et al.^19^ tried to solve the EEG signal compressed perceptual reconstruction problem using Deep Learning (DL) approach. These authors used Stacking Denoising Auto Encoder (SDAE)^20^ with three hidden layers, combined with the end-to-end feature of deep learning, to achieve SDAE-based compressed perceptual reconstruction. Given the high computational cost of BCI signal reconstruction using traditional CS technology, Ritu Ranjan Shrivastwa et al.^21^ proposed a reconstruction framework based on Convolutional Neural Network (CNN) to reconstruct the pinnacle signal that is highly compressed by CS technology and obtained a good reconstruction effect. Also, Hongpo Zhang et al.^22^ proposed a new neural network model, referred to as CSNet, which combines CNN and a long/short-term memory network. With the ascending dimension signal measured by the ECG signal as input and the complete ECG signal as an output, the reconstruction speed is at least 45 times faster than the traditional ECG compression sensing reconstruction algorithm. In another study, YunFei Cheng et al.^23^ proposed a non-iterative real-time reconstruction model of compressed sensing physiological signals based on a residual network (ResNet), referred to as the Compressed Sensing-Dilated Residual Network (CS-DRN). This method trains a network model for compressed sensing reconstruction based on a large number of physiological signal data. Experiments show that the model has good reconstruction accuracy and reconstruction time. Sobhan Sheykhivand^24^, Put forward a kind of CS theory and deep neural network (DNN) combining two stages of driving fatigue automatic classification system, the network structure includes seven convolution layer and three long short-term memory layer, the proposed method of driver fatigue two stages classification accuracy improved, can be used for driver fatigue two stage classification.

Currently, deep learning has made some progress in the processing of EEG signals, such as using models such as recurrent neural network (RNN)^25^ and CNN^26^ for EEG signal reconstruction. However, these methods also have some limitations. RNN models may suffer from the problems of gradient vanishing or explosion when processing long sequences, making the model difficult to train and optimize. In addition, RNN models need to consider the time order of the sequence, so the EEG signals need to be time-aligned, which may introduce additional errors^27^. CNN models need to segment and downsample the signals when processing EEG signals, which may result in loss and distortion of signal information^28^. Moreover, traditional CNN models typically use two-dimensional convolution operations, while EEG signals are one-dimensional, which may lead to inappropriate bias in processing EEG signals.

To address these limitations, this study proposes a fast non-iterative EEG reconstruction algorithm based on an improved ResNet and one-dimensional dilation convolution. Specifically, the improved ResNet deepens the residual block, allowing the network to learn more complex feature representations, and increasing the block depth can increase the network's nonlinearity, enabling the network to better fit complex EEG signal data and improve reconstruction accuracy. One-dimensional dilation convolution can expand the receptive field of the convolution kernel and effectively reduce the number of parameters during model training, thereby reducing computational costs and time. At the same time, compared with the traditional algorithm, the reconstruction accuracy still remains good in the case of low compression. Simulation experiments on the BCI IV-2a and BCI III-a competition open data sets show that the proposed network model can achieve the best reconstruction accuracy under some compression ratios.

## Related theory

### Compressed sensing

**Figure 1:**
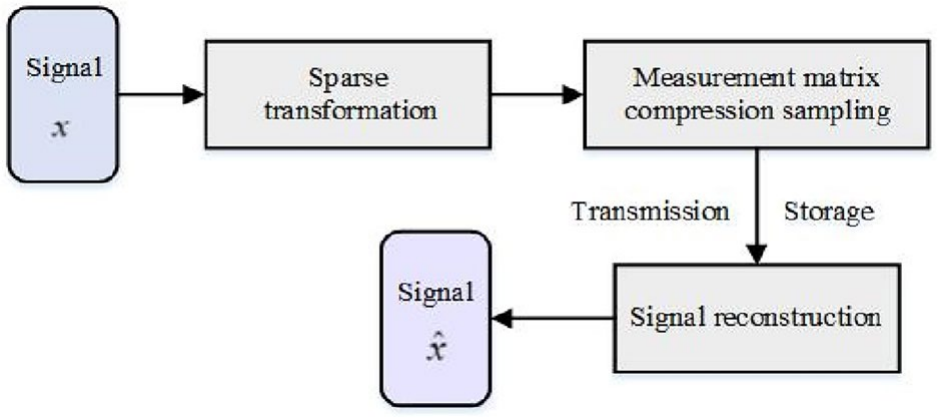
Framework of compressed sensing.

Compressed sensing theory means that when the original signal itself is sparse, or when the signal is sparse on the transform orthogonal basis, the random stationary observation matrix can be used for compression sampling based on spatial transformation. a compressed signal which keeps the information of the original signal and the length of the signal is much smaller than that of the original signal is obtained, and then the original signal is accurately reconstructed by solving the iterative optimization problem. Compressed sensing theory includes sparse transformation, measurement matrix design and signal reconstruction. The basic structure is shown in Fig. 1.

Compressed sensing theory is aimed at sparse signals, so the non-sparse original signal ***x*** can be represented as sparse signal ***s*** by a transform orthogonal basis in this transform domain before it can be processed by compressed sensing method. The common sparse transforms are wavelet transform, Fourier transform and discrete cosine transform. The sparse transformation formula is as follows:

1 $$x=\Psi s$$

where $\boldsymbol{\Psi }$ denotes the sparse basis, which is the orthogonal basis of transforming the original signal ***x*** to the sparse signal ***s***; ***s*** is the signal where the sparsity is ${\varvec{K}}({\varvec{K}}\ll {\varvec{N}})$ signal, and the sparsity ***K*** means that there are only ***K*** non-zero values in ***s***.

The data compression expression can be obtained directly through the compressed sensing theory, and the intermediate step of obtaining N-dimensional signal ***x*** is omitted. The linear observation model is as follows:

2 $${\varvec{y}}=\boldsymbol{\Phi }{\varvec{x}}=\boldsymbol{\Phi }\boldsymbol{\Psi }{\varvec{s}}=\boldsymbol{\Theta }{\varvec{s}}$$

where ***x*** is the signal of ${\varvec{N}}\times 1$ dimension, ***y*** is the signal of ${\varvec{M}}\times 1$ dimension, and $\boldsymbol{\Phi }$ is the observation matrix of ${\varvec{M}}\times {\varvec{N}}({\varvec{M}}\ll {\varvec{N}})$ dimension.

Compressed sensing is used to reconstruct the signal $\widehat{{\varvec{s}}}$ from the sampled compression vector ***y***, and then the reconstructed signal $\widehat{{\varvec{x}}}$ is derived by inverse transformation as follows:

3 $$\widehat{{\varvec{x}}}=\boldsymbol{\Psi }\widehat{{\varvec{s}}}$$

However, in order to correctly reconstruct the sparse signal $\widehat{{\varvec{s}}}$ based on the compressed signal ***y***, the following two conditions must be satisfied.

I. The dimension of y is ${\varvec{M}}={\varvec{O}}({\varvec{K}}\times {\varvec{l}}{\varvec{g}}{\varvec{N}})$, where ***O*** is the operator that computes the complexity.

II. The observation matrix $\Phi$ satisfies the restricted equidistance property criterion, that is, there exists a bounded equidistance constant ${\delta }_{k}\in ({0,1})$. Therefore, for any signal *x* with sparsity *K*, the following equation holds:

4 $$\left(1-{{\varvec{\delta}}}_{{\varvec{k}}}\right){\Vert {\varvec{x}}\Vert }_{2}^{2}={\Vert \boldsymbol{\Phi }{\varvec{x}}\Vert }_{2}^{2}=\left(1+{{\varvec{\delta}}}_{{\varvec{k}}}\right){\Vert {\varvec{x}}\Vert }_{2}^{2}$$

If the above two conditions are satisfied, the solution process can be transformed into an NP-hard problem for the parametric number ${{\varvec{l}}}_{0}$:

5 $${\varvec{m}}{\varvec{i}}{\varvec{n}}{\Vert {\boldsymbol{\Psi }}^{{\varvec{T}}}{\varvec{x}}\Vert }_{0}\boldsymbol{ }{\varvec{s}}.{\varvec{t}}.\boldsymbol{ }{\varvec{y}}=\boldsymbol{\Theta }{\varvec{s}}=\boldsymbol{\Phi }\boldsymbol{\Psi }{\varvec{s}}$$

Eventually, the signal $\widehat{{\varvec{s}}}$ with sparsity ***K*** is found by ***y***. Then, the reconstructed signal $\widehat{{\varvec{x}}}$ can be found according to Eq. (3). The above problem can be transformed into a convex optimization problem, which can be solved by convex optimization algorithm or greedy algorithm, which is widely used at present. Greedy algorithms mainly include OMP algorithm^29^, Compressed Sampling Matching Pursuit (CoSaMP) algorithm^30^ and so on. However, no matter which kind of compression and reconstruction algorithm, there are requirements for the sparsity of the signal, and the signal needs to be processed in the early stage. If the signal sparsity is unknown, it is necessary to estimate the signal sparsity in the early stage. In addition, due to the iterative operation of each reconstruction, the amount of computation is large and the process is complex, these algorithms are difficult to be applied to a large number of signal reconstruction. In the process of compression and reconstruction, the reconstruction effect of these algorithms is unstable, which is not conducive to large-scale signal compression and reconstruction.

### Residual neural network

Residual neural network was proposed by He et al.^31^ of Microsoft Research, who discovered the degeneration phenomenon and invented the shortcut connection for the degeneration phenomenon. This network effectively solved the problem of training difficulties caused by too deep neural networks.

**Figure 2:**
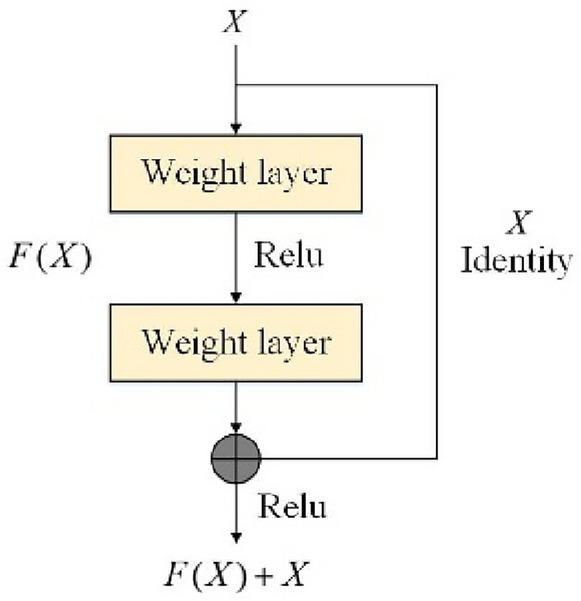
Basic structure of residual block.

In a normal neural network, the output of each layer is connected to the input of the next layer. If enough data is available, accuracy usually increases with the number of layers and neural network parameters. However, as the number of layers increases, the accuracy of the neural network saturates or begins to decline. In this situation, problems such as vanishing gradient and overfitting can cause the initial layers to fail to adjust well. Therefore, shallower networks in a normal neural network seem to be better than deeper ones. ResNet, on the other hand, solves this problem using skip connections or residual connections to skip additional layers to achieve the accuracy of shallow networks^32^. The residual blocks are shown in Fig. 2.

The residual block is divided into two parts: the direct mapping part and the residual part. $X$ on the right side of the figure is the input itself, which is the direct mapping part. The left side of the figure $F(x)$ is the residual part, which consists of two convolution operations and the activation ReLU function, where $F\left(x\right)+X$ represents a complete residual block.

In this paper, a deep learning network structure is designed according to the idea of the original ResNet model to complete the compression and reconstruction of one-dimensional EEG signals.

## Methods

### EEG signal reconstruction process

**Figure 3:**
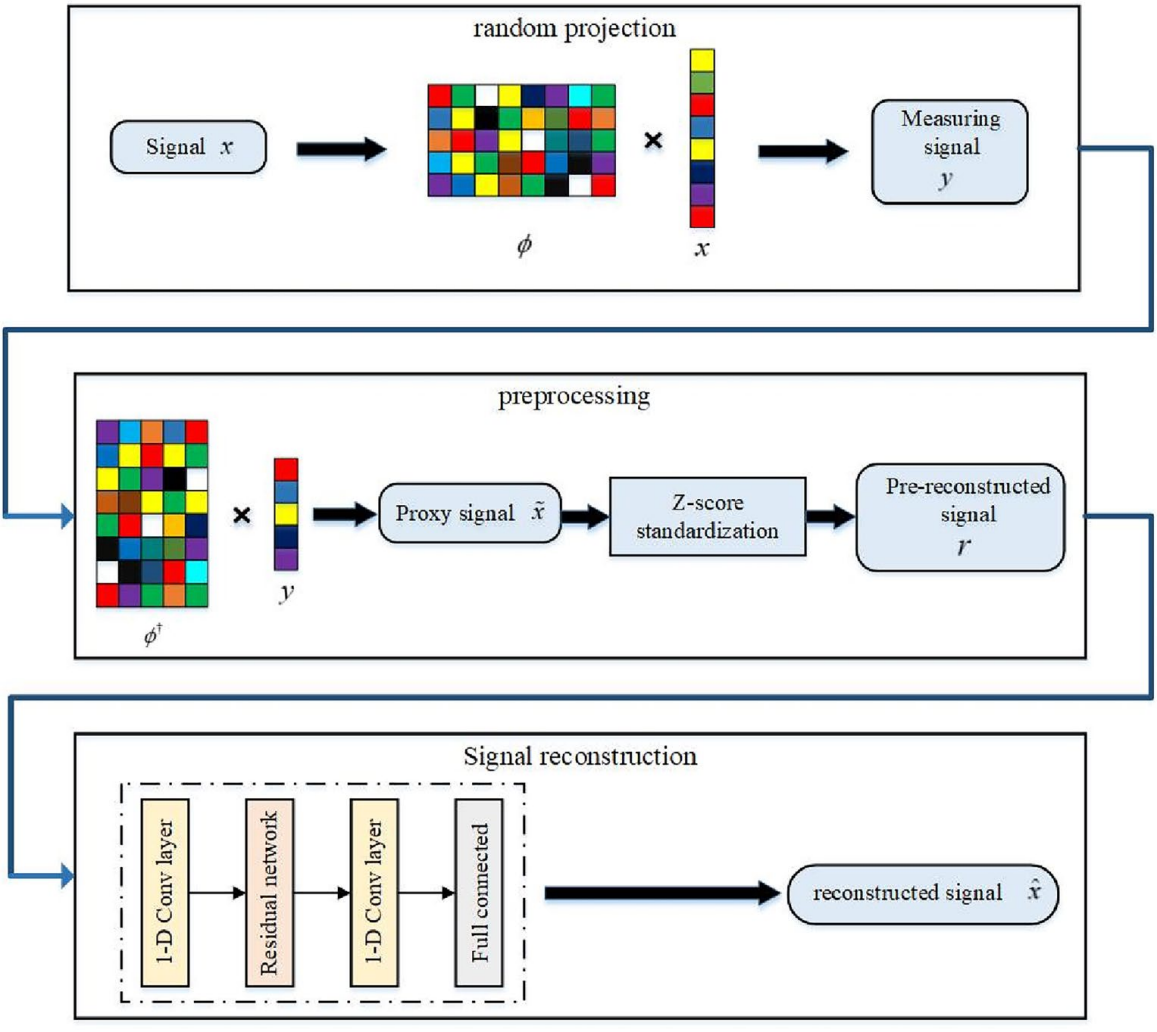
Flow chart of EEG signal compression sensing and reconstruction based on deep learning.

In deep learning, a neural network is a general function approximator that can learn to approximate any continuous function with enough training data, which makes it possible to use deep learning methods to implement CS reconstruction. Therefore, this paper uses an improved ResNet to “learn” an inverse operation similar to CS reconstruction rather than solving the inverse problem of CS reconstruction. The flowchart of EEG perceptual signal compression and deep learning-based signal reconstruction of this paper is shown in Fig. 3.

In the first step, random projection is performed. The EEG signal is CS acquired and compressed, and the original EEG signal data set is represented as $X=\{{x}^{\left(1\right)},\dots ,{x}^{\left(i\right)}{,\dots ,x}^{\left(l\right)}\}$. In this function, ${x}^{(i)}\in {R}^{1\times n}$ denotes the i-th EEG signal, ${x}^{(i)}=({x}_{1}^{\left(i\right)},{x}_{2}^{\left(i\right)}{,\dots ,x}_{n}^{\left(i\right)})$ with $n$ sampling points. The compressed EEG signal is expressed as $Y=\{{y}^{\left(1\right)},\dots ,{y}^{\left(i\right)}{,\dots ,y}^{\left(l\right)}\}$, where ${y}^{(i)}\in {R}^{1\times m}$ denotes the i-th measurement signal, ${y}^{(i)}=({y}_{1}^{\left(i\right)},y{,\dots ,y}_{m}^{\left(i\right)})$ with $m$ sampling points. The mapping of the compressed EEG signal $Y$ to the original EEG signal $X$ can be regarded as an approximate linear mapping, i.e., $Y=\Phi X$, where $\Phi \in {R}^{m\times n}$ (m < n) is a linear mapping matrix. The CS acquisition process of the i-th EEG signal is expressed as follows:

6 $${y}^{(i)}={\upphi }_{m\times n}{x}^{(i)}$$

In the second step, preprocessing. For the measured values $Y=\{{y}^{\left(1\right)},\dots ,{y}^{\left(i\right)}{,\dots ,y}^{\left(l\right)}\}$, where ${y}^{(i)}$ has m sampling points (m < n). Here, the compressed measuring signal ${y}^{(i)}$ are multiplied to the pseudo-inverse of the measurement matrix ${\upphi }_{m\times n}$ to obtain the proxy signal. The specific process is expressed as Eq. (7):

7 $${\widetilde{x}}^{(i)}={\phi }_{m\times n}^{\dagger}{y}^{(i)}$$

where ${\widetilde{x}}^{(i)}$ denotes the proxy signal having the same size as the original signal dimension, with n samples and ${\upphi }_{m\times n}^{\dagger}$ is the pseudo-inverse of the measurement matrix ${\upphi }_{m\times n}$. Using this approach, the known measurement matrix and the compressed signal can be well used for learning and thus for accurate reconstruction. To speed up network training, the proxy signals need to be z-score normalized to transform the data to a mean of 0 and standard deviation of 1 so that $\widetilde{X}=\left\{{\widetilde{x}}^{\left(1\right)},\dots ,{\widetilde{x}}^{\left(i\right)},\dots ,{\widetilde{x}}^{\left(l\right)}\right\}$ follows the standard normal distribution. Under the projection of the measurement matrix, the range of each signal value changes, and normalization helps normalize the values by compressing the original data values to a smaller range, effectively improving the convergence rate of gradient descent (GD). The standardization process is as Eq. (8):

8 $${r}^{(i)}=\frac{{\widetilde{x}}^{(i)}+{\widetilde{x}}_{\mu }^{(i)}}{{\widetilde{x}}_{\sigma }^{(i)}},i={1,2}\dots ,l$$

where ${\widetilde{x}}^{(i)}$ is the proxy signal, ${\widetilde{x}}_{\mu }^{(i)}$ is the mean of the proxy signal, ${\widetilde{x}}_{\sigma }^{(i)}$ is the standard deviation of the proxy signal, and ${r}^{(i)}$ is the normalized data of the proxy signal.

In the third step, the signal is reconstructed. In this paper, a modified residual-based network is used to fit the nonlinear mapping relationship from the preprocessed signal ${r}^{(i)}$ to the original signal $x$. The reconstructed signal is represented by ${\widehat{x}}^{(i)}$ (Eq. 9):

9 $${\widehat{x}}^{(i)}=H\left({r}^{\left(i\right)}\right),i={1,2},\dots ,l$$

where *H* denotes the improved ResNet model.

In reconstructing the network, this paper divides the EEG signal dataset into training and test sets and follows the label consistency criterion. The method is as follows:

Here, the training set is as follows: ${D}_{train}=\{\left({r}^{\left(1\right)},{x}^{\left(1\right)}\right),\dots ,\left({r}^{\left(i\right)},{x}^{\left(i\right)}\right),\dots ,({r}^{\left(q\right)},{x}^{\left(q\right)})\}$, the training set includes the preprocessed signals of $q$ sets of measurements and the corresponding labels. Unlike labeling the categorization network, the label here refers to the raw EEG signal, rather than merely some categorization categories. Similarly, the test set is expressed as: ${D}_{tesr}=\{\left({r}^{\left(1\right)},{x}^{\left(1\right)}\right),\dots ,\left({r}^{\left(i\right)},{x}^{\left(i\right)}\right),\dots ,({r}^{\left(s\right)},{x}^{\left(s\right)})\}$, the test set includes the preprocessed signals of the $s$ sets of measurements and the corresponding labels, $q+s=l$. To realize accurate reconstruction, the loss function L is defined as the Mean Square Error (MSE) between the reconstructed signal ${\widehat{x}}^{(i)}=({\widehat{x}}_{1}^{\left(i\right)},{\widehat{x}}_{2}^{(i)},{\dots ,\widehat{x}}_{n}^{(i)})$ and the original signal ${x}^{(i)}=({x}_{1}^{\left(i\right)},{x}_{2}^{(i)},{\dots ,x}_{n}^{(i)})$, expressed as follows:

10 $$L=\frac{1}{l}\sum_{i=1}^{l}{\Vert {\widehat{x}}^{(i)}-{x}^{(i)}\Vert }_{2}^{2}$$

The nonlinear mapping relationship between the preprocessed signal and the original signal is learned, by training the improved ResNet model. The loss function minimizes the error between the original and reconstructed signals. The GD algorithm is used to optimize the network weight parameters, so that the result of the loss function is as small as possible so that the reconstructed EEG data and the original data are the same as possible.

### Structure of the improved ResNet model

**Figure 4:**
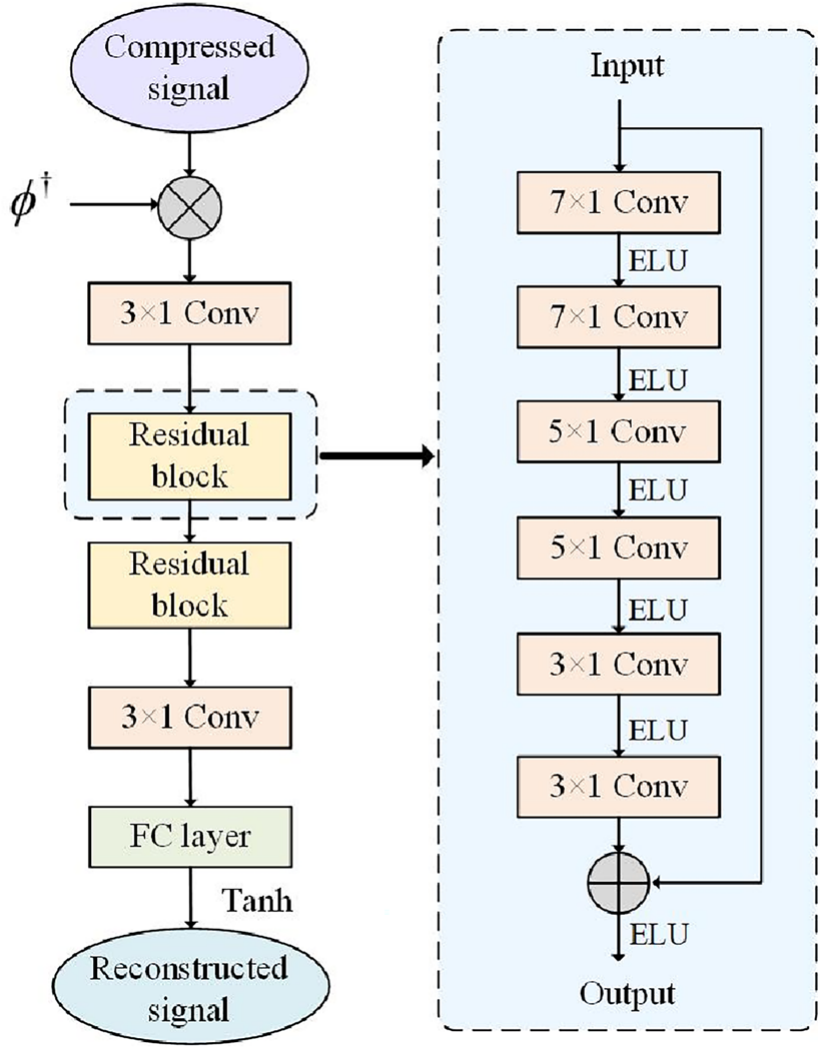
Improved residual network model structure.

The structure diagram of the improved ResNet model is shown in Fig. 4:

This structure is named CS-ResNet in this paper. CS-ResNet contains two residual blocks. Each residual block contains six one-dimensional dilated convolution layers, where the input and output dimensions of the residual blocks are the same, both being characteristic graphs of 16 channels. To ensure that the inputs and outputs of residual blocks in the residual learning network can be added, the numbers of convolution kernels in the six one-dimensional dilated convolution layers in the residual block are 32, 64, 128, 64, 32, and16, respectively, and the corresponding convolution kernels are $7\times 1$, $7\times 1$, $5\times 1$, $5\times 1$, $3\times 1$, and $3\times 1$, respectively, and the dilated rate of each convolution kernel is set to 2. According to the size and dilated rate of the current convolution kernel, an appropriate filling value is set in each convolution layer to keep the size of the feature graph constant. The Exponential Linear Unit (ELU) function^33^ is used as the activation function after each layer of convolution in the residual block. ELU incorporates Sigmoid and ReLU. Also, the left side of its function curve has soft saturation, which enables ELU to be more robust to input changes or noise. On the other hand, the right side has no saturation, which makes ELU mitigate gradient vanishing. As a result, the network convergence is realized more easily, and the model training is sped up.

**Figure 5:**
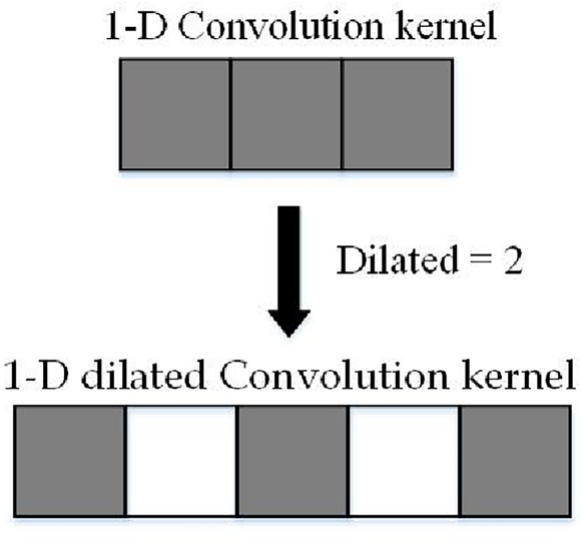
One-dimensional dilation convolution is used in this paper.

The one-dimensional dilated convolution is shown in Fig. 5.

Compared with one-dimensional convolution, one-dimensional dilation convolution introduces a hyperparameter called Dilation Rate, which injects voids into the standard convolution kernel to control the number of null values 0 in the convolution kernel. Using dilation convolution allows for increasing the perceptual field of the convolution and reducing the number of model parameters, resulting in improved model performance. The size of the convolution window after expansion can be calculated from Eq. (11):

11 $${{\varvec{h}}}^{\boldsymbol{^{\prime}}}={\varvec{h}}+({\varvec{h}}-1)({\varvec{d}}-1)$$

where ${{\varvec{h}}}^{\boldsymbol{^{\prime}}}$ is the size of the one-dimensional dilation convolution window, ***h*** is the size of the original convolution window, and ***d*** is the dilation rate.

After processing, the signal is first converted into a feature map of 16 channels by a one-dimensional dilation convolution layer with 16 convolution kernels and $3\times 1$ convolution kernels. After convolution, the ELU activation function is used, followed by two residual blocks with the same structure, a one-dimensional dilation convolution layer with 1 convolution kernel, and $3\times 1$ convolution kernels. Finally, the reconstructed EEG signal is output by a full connection layer.

## Experimental analysis

### Experimental data set selection and processing

The experimental data were obtained from the public dataset BCI IV-2a, consisting of EEG data from nine subjects. In the brain-computer interface experiment, subjects perform a motor imagery task based on arrows appearing on the computer screen pointing left, right, down or up (corresponding to the left hand, right hand, foot or tongue, respectively). In these experiments, EEG was recorded using 22 Ag/AgCl electrodes (3.5 cm distance between electrodes), and EEG signal data were collected from 22 channels. All signals were recorded as monopolar, with the left mastoid as a reference and the right mastoid as a grind. The signal is sampled with 250 Hz and bandpass filtered between 0.5 and 100 Hz. The amplifier’s sensitivity is set to 100 ${\upmu{\rm V}}$, and an additional 50 Hz trap filter suppresses line noise.

In the experiments of this paper, since the compressed sensing is segmented for EEG signal acquisition, the whole EEG signal is intercepted into signal frames of only 2 s in length for each period, and the length of each signal frame is obtained as ${\varvec{N}}=500$. Among the intercepted EEG signals, 80% are randomly selected as the training set and 20% as the test set. To obtain a compressed signal of length ***M***, the EEG signal needs to be projected to a lower dimension using a measurement matrix. In this study, the Gaussian random matrix^34^ and sparse binary matrix^35^ are selected to compress the signal. Afterward, it is proved through experiments that these two different measurement matrices have little effect on the reconstruction accuracy of the compressed signal. However, the Gaussian random matrix has a large memory footprint and high consumption of resource operations, which will be greatly limited in practical applications. In addition, the sparse binary matrix is sparse, and the number of element values in the matrix is 1 or 0, the number of 1’s is far less than 0. As a result, the matrix multiplication calculation only needs to perform an integer addition operation when the element value is 1, thereby reducing the number of operations and making it easy to implement in hardware. Briefly, this paper selects the sparse binary matrix as the measurement matrix. The compression ratio is defined as:

12 $$CR=\frac{M}{N}$$

In this paper, we construct measurement matrices with N = 500 and M = 50, 100, …, 400, 450 with corresponding compression ratios CR = 10%, 20%, …, 80%, 90%.

### Experimental data set selection and processing

In this paper, we use the percent root mean squared difference (PRD) to evaluate the reconstruction accuracy of EEG signals:

13 $${\varvec{P}}{\varvec{R}}{\varvec{D}}=\frac{{\Vert \widehat{{\varvec{x}}}-{\varvec{x}}\Vert }_{2}}{\Vert {\varvec{x}}\Vert }\times 100\boldsymbol{\%}$$

where ***x*** and $\widehat{{\varvec{x}}}$ denote the original signal and the reconstructed signal, respectively. In this equation, a smaller value of PRD means a higher reconstruction accuracy. This paper uses the average reconstruction time of each signal frame to reflect the reconstruction speed of the reconstruction algorithm.

### Comparison of reconstruction algorithms

The advantages of the proposed network model in brain signal reconstruction were verified by comparing the experimental results with the traditional iterative compressed sensing reconstruction OMP and CoSaMP. In addition, the existing deep learning compressed sensing reconstruction algorithms are compared: RNN, CNN, CSNet, and CS-DRN. The computer configuration used for model training in this paper is shown in Table 1. The processor is AMD Ryzen5 5600H with a main frequency of 3.3 GHz, the graphics card is NVIDIA Geforce RTX 3050, and the video memory is 4 GB. In the above algorithms, the traditional iterative compressed sensing reconstruction algorithm is based on the MATLAB platform. Also the deep learning compressed sensing reconstruction algorithm is implemented by python language and PyTorch framework. The programming environment is PyCharm, the deep learning framework is PyTorch 1.10.2, and the programming language is Python 3.7.11. In the model training, the Adam optimizer^36^, with a learning rate of 0.001 and a batch size of 64, is used to optimize the model. Comparing the results of EEG reconstruction with the above algorithms proves that the model structure proposed in this paper is effective.

**Table 1:** Computer configuration.

Configuration	Specification
Processor	AMD Ryzen5 5600H
Processor Frequency	3.3 GHz
Graphics Card	NVIDIA GeForce RTX 3050
Video Memory	4 GB
Programming Environment	PyCharm
Deep Learning Framework	PyTorch 1.10.2
Programming Language	Python 3.7.11

Table 2 shows the PRD on the BCI IV-2a public dataset. From the table, it can be seen that the PRD values for OMP and CoSaMP are very large, indicating poor quality of reconstructed EEG signals, especially with a compression ratio between 10 to 50%, where the reconstruction accuracy drops sharply. Within this compression ratio range, it is difficult to effectively reconstruct EEG signals because when the compression ratio is lower, the sparsity of the signal is also lower, meaning the coefficients of the signal in some transform domain are more dispersed and difficult to represent as a sparse vector. This will significantly increase the error when using sparse representation algorithms for reconstruction, leading to a sharp increase in reconstruction error. In contrast, reconstruction algorithms based on deep learning perform better than OMP and CoSaMP algorithms at various compression ratios. Due to the fact that the mean squared error percentage values between 10 to 30% are much larger than those between 40 to 90%, it is inconvenient to compare the average values of the overall compression ratio. Therefore, the mean squared error percentage values between 10 to 30% and 40% to 90% are separately compared.

**Table 2:** Percentage of root mean square difference of seven algorithms on BCI IV-2a data set under different compression ratios.

Compression ratio (%)	Traditional iterative algorithm			Deep learning % algorithm			
	OMP	CoSaMP	RNN	CSNet	CS-DRN	MFF-SE	CS-ResNet
90	12.3257	11.3568	1.3272	0.8691	0.6063	0.5831	**0.5485**
80	13.2098	12.2354	1.7395	1.0125	0.6133	0.6256	**0.5976**
70	14.1386	13.6523	1.7847	1.1659	0.6973	0.7234	**0.6198**
60	15.5743	14.3578	1.8024	1.3049	0.7971	0.9158	**0.6506**
50	39.5515	37.8964	2.0682	1.3238	0.8316	1.2445	**0.7996**
40	73.1031	78.8149	2.1524	1.9417	1.1811	1.5562	**1.0016**
30	85.8574	93.1451	**2.1698**	4.7611	4.0911	4.2347	3.9906
20	93.8213	98.9715	**16.4000**	24.2442	23.0156	22.5815	20.1886
10	100.6494	103.9097	**29.0360**	33.1659	38.0639	32.6535	30.2299

Significant values are in bold.

**Figure 6:**
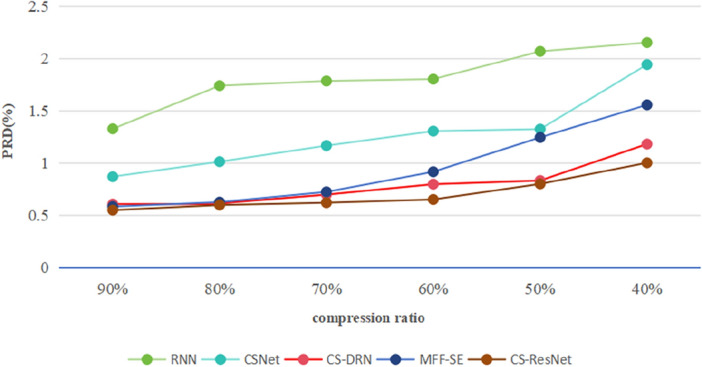
PRD of five deep learning algorithms 40–90%.

As shown in Fig. 6, The CS-ResNet proposed in this chapter outperforms the CSNet, CS-DRN, and MFF-SE^37^ networks in terms of reconstruction accuracy at all compression ratios. This is because the CS-ResNet proposed here can learn the complex non-linear features in the signal and can better deal with the noise and distortion problems in the signal. At the same time, one-dimensional expansion convolution can increase receptive field, maintain input length and capture multi-scale letter when processing EEG signals. This allows it to improve the performance of the model when dealing with EEG signal sequences with time dependence, so that CS-ResNet has the best reconstruction accuracy.

**Figure 7:**
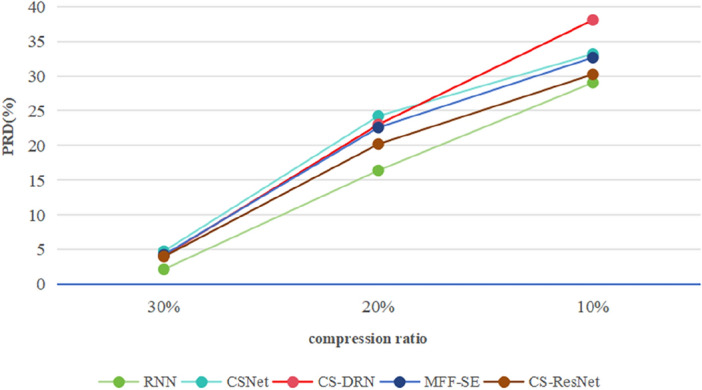
PRD of five deep learning algorithms 10–30%.

In Fig. 7, when the compression ratio is 40% to 90%, the average reconstruction accuracy of CS-ResNet is about 0.57% higher than that of CSNet, 0.08% higher than that of CS-DRN, and 0.24% higher than that of MFF-SE. In Fig. 7, with the compression ratio of 10% to 30%, the average reconstruction accuracy of CS-ResNet is about 1.29% higher than CSNet, 1.79% higher than CS-DRN, and 1.69% higher than MFF-SE. Compared to the RNN algorithm, the CS-ResNet proposed in this chapter performs better in terms of reconstruction accuracy at a compression ratio between 40 to 90%. The average reconstruction accuracy of CS-ResNet is about 1.11% higher than that of RNN. However, at a compression ratio between 10 to 30%, the average reconstruction accuracy of CS-ResNet is lower than that of RNN. This is because at low compression ratios, RNN has stronger memory capabilities, with memory units that can store and transmit information from previous time steps, and can make full use of temporal correlations to model and predict information from compressed signals with less information, resulting in higher reconstruction accuracy. In contrast, CS-ResNet may require more parameters to adapt to the data when facing compressed signals with less information, resulting in high computational complexity and a higher risk of overfitting, which ultimately affects reconstruction accuracy.

**Figure 8:**
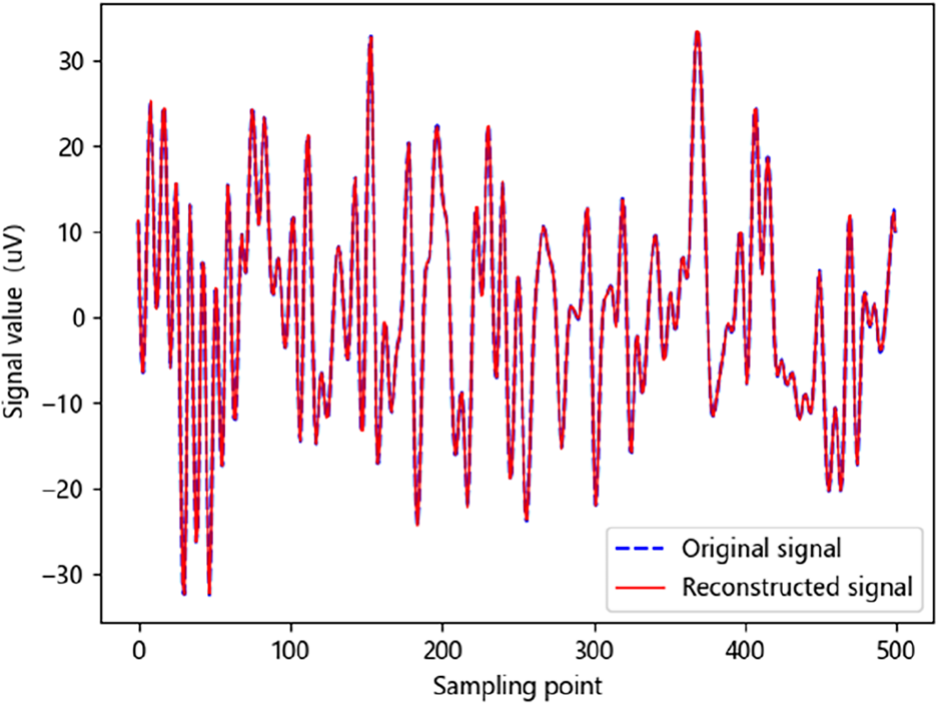
Original signal and reconstructed signal with compression ratio of 70%.

Figure 8 shows the original and reconstructed signal of CS-ResNet at a compression ratio of 70%. The blue dotted line represents the original signal, and the red solid line represents the reconstructed signal. The horizontal axis of the graph represents the sampling points, and the vertical axis represents the signal value. It can be clearly seen from the graph that the original signal curve and the reconstructed signal curve fit very well, which proves that the quality of the reconstructed EEG signal generated by the CS-ResNet reconstruction model is very good, and the reconstruction accuracy is quite high.

Table 3 shows the average reconstruction time of seven algorithms at different compression ratios. It can be seen from Table 3 that the average reconstruction time of traditional iterative algorithms is much higher than that of deep learning algorithms. Obviously, this cannot meet the requirement of fast reconstruction of EEG signals. The average reconstruction time of deep learning algorithms is basically around 1 ms. This is because after the reconstruction model is trained, the compressed signal input only needs to perform several matrix–vector multiplications to complete the reconstruction of EEG signals, so the reconstruction speed is very fast. Among these deep learning algorithms, the CS-ResNet proposed in this paper is slightly better and can better meet the requirement of fast reconstruction. In addition, it can be seen from the table that the average reconstruction time of OMP algorithm and CoSaMP algorithm gradually decreases as the compression ratio decreases, while the average reconstruction time of deep learning algorithms does not change with the decrease of compression ratio. This is because the parameter sharing mechanism of the convolution layer allows the convolution kernel to extract features by translation, so the number of parameters of the convolution kernel is not affected by the signal length.

**Table 3:** Average reconstruction time of seven algorithms on BCI IV-2a data set under different compression ratios.

Compression ratio (%)	Traditional iterative algorithm			Deep learning ms algorithm			
	OMP	CoSaMP	RNN	CSNet	CS-DRN	MFF-SE	CS-ResNet
90	686.25	812.59	0.99	1.20	1.90	2.32	**0.98**
80	671.82	718.73	1.10	0.99	2.00	2.32	**0.97**
70	640.64	660.25	1.20	1.10	2.20	2.40	**0.97**
60	637.53	703.14	0.99	0.99	2.00	2.20	**0.99**
50	625.24	640.66	0.99	1.10	2.50	2.32	**0.98**
40	453.16	468.74	1.00	1.00	2.00	2.32	**0.97**
30	419.26	437.54	0.99	0.99	1.90	2.40	**0.99**
20	388.95	398.68	1.00	1.10	2.00	2.32	**0.98**
10	339.55	353.59	1.00	1.00	2.10	2.32	**0.98**

Significant values are in bold.

**Figure 9:**
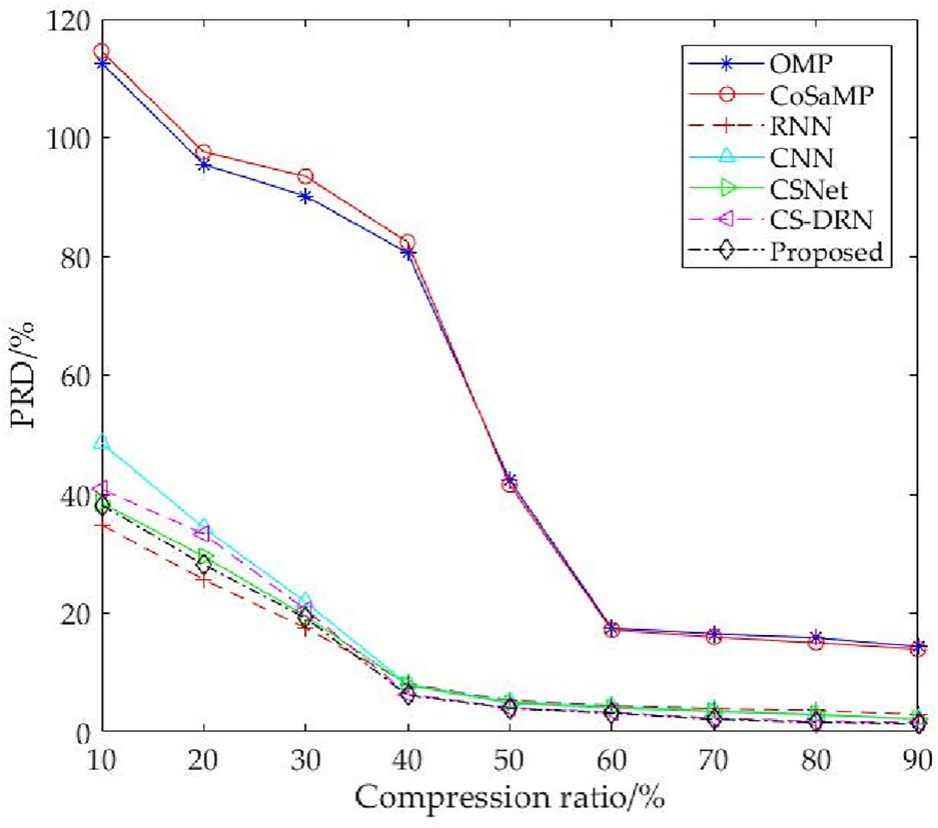
PRD under different compression ratios on BCI III-a datasets.

Figure 9 shows the reconstruction errors of seven compressed sensing reconstruction algorithms on BCI III-a data sets under different compression ratios. It can be seen from the figure that the PRD values of traditional iterative algorithms OMP and CoSaMP are much higher than those of other algorithms under each compression ratio. All the algorithms based on deep learning decrease with the increase in compression ratio, and the PRD values of the proposed method are better than those of CNN, CSNet, and CS-DRN under different compression ratios. Compared with the RNN algorithm, this method’s PRD values are better than RNN in the compression ratio range of 40% to 90%. This result is consistent with the conclusion obtained from the BCI IV-2a data set.

**Figure 10:**
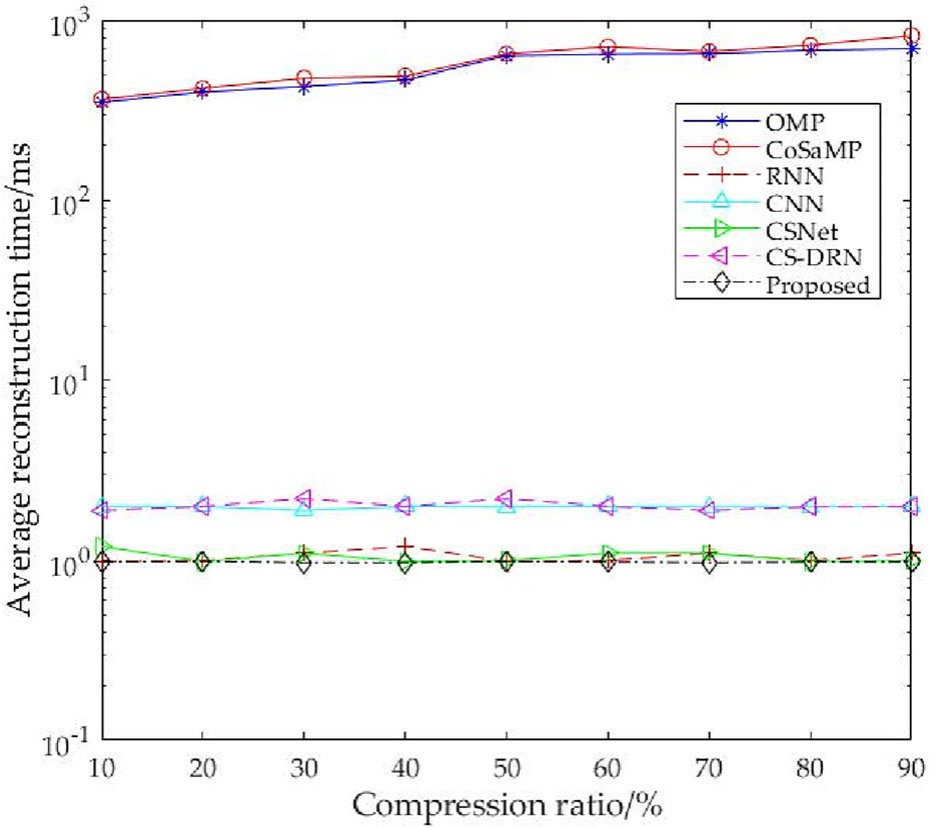
Average reconstruction time under different compression ratios on BCI III-a dataset.

Figure 10 shows the average reconstruction time of seven compressed sensing reconstruction algorithms on BCI III-a data sets under different compression ratios. It can be seen from the figure that the average reconstruction time of the deep learning algorithm is about two orders of magnitude less than that of traditional iterative algorithms. In contrast, the average reconstruction time of this method is better than that of other reconstruction algorithms, which reflects the rapidity. This finding is also consistent with the conclusion obtained from the BCI-IV-2a data set.

In this paper, compressed sensing reconstruction experiments are carried out on BCI IV-2a data sets and BCI III-a data sets. The experimental results show that this method is superior to other algorithms and can realize the rapid reconstruction of EEG signals with high precision. This is because the CS-ResNet proposed here can learn the complex nonlinear features in the signal and can better deal with the noise and distortion problems in the signal. Secondly, the residual network is a low-complexity network structure that can effectively reduce the number of network parameters and computational complexity, thus reducing the time and resource cost of model training and inference. so CS-ResNet has the best reconstruction accuracy. Maintaining high signal quality while reducing computational and transmission overhead to meet the requirements of real-time processing. Additionally, one-dimensional dilated convolutions can reduce the number of parameters and computational time by sparsely applying convolutional kernels over the input sequence. Compared to traditional convolution operations, dilated convolutions increase the receptive field while maintaining the length of the input sequence, thereby enhancing the reconstruction accuracy of EEG signals. However, at low compression rates, our network is not optimal, which is an aspect that requires further research and improvement.

## Conclusion

This paper proposes a CS-ResNet model for compressed sensing reconstruction of EEG signals. The improved residual network can alleviate the gradient vanishing problem caused by the increasing depth of the neural network, improve the network's learning ability and training efficiency. The one-dimensional dilated convolution can expand the receptive field of the convolution kernel, effectively reduce the number of parameters during model training, and extract feature information of EEG signals. This allows for fast and accurate reconstruction of EEG signals. Compared with traditional iterative compressed sensing reconstruction algorithms, the proposed method in this paper can achieve high-precision reconstruction of EEG signals without iteration. The reconstruction speed is two orders of magnitude faster than the traditional iterative compressed sensing reconstruction algorithm. The proposed method has better reconstruction accuracy and speed than deep learning algorithms CNN, CSNet, and CS-DRN. For the RNN algorithm, this method's reconstruction accuracy is better than that of RNN when the compression ratio is between 40 and 90%, and the reconstruction time is also better than RNN. Since this method has broad application prospects in future EEG remote monitoring systems, we need to conduct further research to achieve high-precision reconstruction of EEG signals under low compression ratios.

## Data Availability

The datasets generated and/or analyzed during the current study are not publicly available due to project privacy concerns but are available from the corresponding author on reasonable request.
